# Time pressure and sleep problems due to thoughts about work as risk factors for future sickness absence

**DOI:** 10.1007/s00420-018-1349-9

**Published:** 2018-08-20

**Authors:** Pia Svedberg, Lisa Mather, Gunnar Bergström, Petra Lindfors, Victoria Blom

**Affiliations:** 1 Division of Insurance Medicine, Department of Clinical Neuroscience, Karolinska Institutet, 171 77 Stockholm, Sweden; 2 Division of Intervention and Implementation Research for Worker Health, The Institute of Environmental Medicine, Karolinska Institutet, Stockholm, Sweden; 3 Department of Psychology, Stockholm University, Stockholm, Sweden; 4 The Swedish School of Sport and Health Sciences, Stockholm, Sweden; 5 Department of Occupational and Public Health Sciences, Centre for Musculoskeletal Research, University of Gävle, Gävle, Sweden

**Keywords:** Sick leave, Sleep, Time pressure, Twins

## Abstract

**Purpose:**

This study investigated whether time pressure or sleep problems due to thoughts about work are associated with future sickness absence (SA) among women and men employed in different sectors, also when adjusting for confounders including familial factors (genetics and shared environment).

**Methods:**

The study sample included 16,127 twin individuals (52% women), aged 19–47 years who in 2005 participated in an online survey including questions regarding time pressure, sleep, work and health. Register data on SA (> 14 days) were obtained from the National Social Insurance Agency and individuals were followed from date of survey response until 12/31/2013. Associations between time pressure, sleep problems due to thoughts about work and future SA were investigated using logistic regression analyses to assess odds ratios (OR) with 95% confidence intervals (CI).

**Results:**

In total 5723 (35%) individuals had an incident SA spell during follow-up. Sleep problems due to thoughts about work were associated with SA in the fully adjusted model (OR 1.22, CI 1.10–1.36). Stratified by sector, the highest estimate was found for state employees (OR 1.54, CI 1.11–2.13). Familial factors did not seem to influence the associations. We found no statistically significant associations between time pressure and SA. No sex differences were found.

**Conclusions:**

Results indicated that sleep problems due to thoughts about work is a risk factor for future SA. This follows previous research showing that sleep length and sleep disturbances, regardless of reason, are associated with SA. But, experiences of work-related time pressure seem to have no effect on SA.

## Introduction

Work disability in terms of sickness absence (SA) is among the most common public health problems with potentially severe health-related, social and economic consequences for individuals, and vast economic and political concerns for societies. The reasons underlying SA are, apart from disease or injury, likely to involve a combination of different factors including stress and strain in the workplace and poor recovery. Previous research has identified and investigated various risk factors for SA, such as being a woman, older age, high job demands, and a low socioeconomic status (Allebeck and Mastekaasa 2004; Mather et al. 2015; Vries de et al. 2017). So far, fewer studies have investigated time pressure, including overtime work, as well as sleep disturbances, despite these having the potential of being important risk factors for SA. This association may relate to disturbed sleep being associated with fatigue, which in turn is associated with sub-optimal health (Åkerstedt et al. 2014).

Sleep disturbances can be symptoms of disease as well as risk factors for ill health. Sleep problems are widely believed to impair health through their various effects on bodily systems, e.g. neuroendocrine, immune and metabolic systems (McEwen and Karatsoreos 2015). Previous studies show that individuals can handle much strain as long as they can recover, during the day and over time, and during sleep, with sleep having powerful restorative properties (Åkerstedt and Nilsson 2003). Psychological strain affects sleep quality and impairs recovery from overall work strain (Winwood and Lushington 2006) and findings suggest that long work hours are associated with reduced sleep quality in a dose–response manner (Nakashima et al. 2011). Moreover, there is an association between work-related everyday sleep disturbances and SA, as compared to those seldom or never reporting such sleep disturbances, with odds ratios varying between 3.22 and 4.26. However, adjustment for health indicators substantially attenuate the association (Westerlund et al. 2008).

Time pressure including overtime work, skipping lunch, and bringing home work, may reduce opportunities for recovery during leisure time and weekends. Previous research indicates that long workhours and overtime work generate a wide range of adverse outcomes including ill-health symptoms (Sparks et al. 1997; van der Hulst 2003). Overtime workers are more likely to be men, white, middle-aged, and they usually have a high level of education and a higher income. Also, being self-employed, working as an independent contractor, having more than one job, and working split/irregular/on-call shifts seem common (Grosch et al. 2006). While overtime work typically involves higher levels of job stress and time pressure, it has also been associated with protecting factors for stress such as increased possibilities to participate in decision making and opportunities to develop special abilities. Consequently, the associations between working hours and health-related measures are complex, particularly for individuals in the higher overtime group (70 + h/week) (Grosch et al. 2006).

So far, findings regarding working hours and health-related outcomes are inconclusive and scarce, particularly for SA. Some studies show that employees working overtime were no more likely to incur adverse physical or mental health, or disability (Allen et al. 2007). Instead, overtime work beyond 50 h a year was associated with a low SA incidence for women and men (Voss et al. 2001), and working overtime also decreased the risk of short-term SA (Laaksonen et al. 2010). Another study has shown associations between overtime and lower SA among both women and men, with women working the most hours being the least sick-listed (Krantz and Lundberg 2006). These women mainly held top-level positions and besides both women and men in such positions seeming to share household duties more evenly, they may also afford and prioritize having someone assisting them with household duties. However, women working overtime due to workaholism, or to meet supervisors’ expectations, have been found to suffer from mental health problems and social isolation. For these women SA facilitated recovery, but also involved isolation and difficulties in returning to work (Verdonk et al. 2008). With women and men typically working in different sectors of the labor market, it seems important to take this into account by investigating sector. Moreover, SA due to mental diagnoses have increased in recent years in Sweden and seemingly such SA also varies between sectors (National Social Insurance Board (Försäkringskassan) 2014) which provides a further rationale for considering occupational sectors when investigating various working conditions and behaviors in relation to SA.

When researching the associations between time pressure, sleep disturbances due to thoughts about work and SA several confounding factors need to be considered. Using a population-based twin-setting including twins sharing their genes and having grown up in the same family, also allows controlling for genetic and shared environmental (familial) confounders. From previous research, it is well known that genetics influence health conditions and behaviors (Kaprio 2000; Plomin et al. 2013), which, in turn, may influence the risk of experiencing stress. Sleep duration and quality (Gasperi et al. 2017; Goel 2017; Åkerstedt et al. 2017) as well as SA are moderately heritable (Gjerde et al. 2013; Svedberg et al. 2012). Thus, it seems reasonable to assume that familial factors influence the association between sleep disturbances due to thoughts about work and SA.

### Aim

The aim was to study whether time pressure and sleep problems due to thoughts about work are associated with future SA among women and men employed in different sectors, also when adjusting for confounders including familial factors.

## Methods

### Study population

**Fig. 1:**
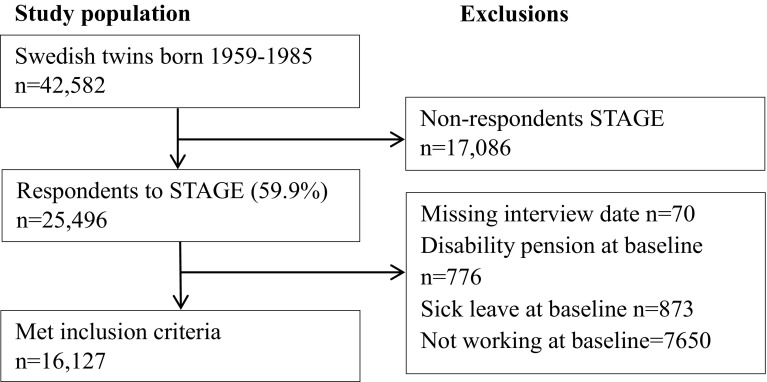
Study population, inclusion and exclusion criteria. *STAGE* the Study of Twin Adults: Genes and Environment

The source population consisted of 25,496 twins born between 1959 and 1985 of the Swedish Twin project Of Disability pension and Sickness absence (STODS) who participated in the Study of Twin Adults: Genes and Environment (STAGE) online survey conducted by the Swedish Twin Registry in 2005 (Lichtenstein et al. 2006). Individuals missing a response date, not working full or part-time, being disability pensioned, or having a SA spell at the time of interview were excluded. The final study sample included 16,127 twins (52.1% women), aged 19–47 (mean 35.4, SD 6.9) (see Fig. 1 for inclusion criteria). Of these, 4267 were complete pairs; 1644 monozygotic (MZ) pairs, 1266 same-sex dizygotic (DZ) pairs and 1273 opposite-sex pairs and 84 with unknown zygosity. Also 7593 single twins were included, i.e. the twin sibling did not respond to STAGE, or were excluded based on the above criteria. For details on zygosity determination in the Swedish Twin Registry, see Lichtenstein et al. (Lichtenstein et al. 2006) and Magnusson et al. (Magnusson et al. 2013).

### Outcome and follow-up time

Sickness absence (SA) data were obtained from the National Social Insurance Agency MicroData for Analyses of Social insurance database (MiDAS) and linked to each individual using the Swedish ten-digit personal identification number. All individuals in Sweden above the age of 16, with an income from work or unemployment benefits, can receive sickness benefits paid by the Social Insurance Agency when disease or injury has caused reduced work capacity. Employees receive sick pay from their employers during the first 14 days after a qualifying day (usually more qualifying days for self-employed) without benefits. SA was operationalized as having at least one incident spell lasting longer than 14 days during follow-up, i.e. between the date of STAGE survey response (varying between 11/01/2004 to 04/21/2006, i.e. an individual responded once during this period) and 12/31/2013. No SA spells during follow-up was used as reference.

### Exposures

Two questions from the Labour Force Survey, Statistic Sweden, and included in STAGE were used. Time pressure was measured asking “Do you have so much to do that you have to work during lunchtime (skip lunch), work overtime or take work home with you?” Response alternatives almost every day, a couple of times a week, once a week, were grouped into “Once a week or more” while response alternatives a couple of days per month or more seldom were grouped into “Twice a month or less”. Responses don’t know/don’t want to answer were coded as missing.

Sleep problems due to thoughts about work was measured with the question “During the last three months; have you had problems sleeping due to thoughts about work?” The response alternatives every day/night or nearly every day/night, a couple of days/nights per week and one day/night a week were added together to “Once a week or more” and a couple of days/nights per month and more seldom or not at all were added together to “Twice a month or less”. Responses don’t know/don’t want to answer were coded as missing.

### Covariates

Age was included as a continuous variable derived by subtracting the date of response to STAGE from the birthdate. Sex was dichotomous (woman/man). The highest level of education was categorized into three groups (1) Elementary school, (2) Vocational school including residential college for adult education, and (3) University degree (including military school and vocational university). Marital status was grouped into married/cohabiting or not.

Main employer was assessed with the question “Who has been your main employer during the past three years?” with the response alternatives: (1) State (2) Municipality (3) County council (4) Private sector (5) Self-employed (6) Other. Don’t know/Don’t want to answer was coded as missing.

The Swedish translation (Sanne et al. 2005) of the Karasek and Theorell (Karasek and Theorell 1990) questionnaire was used to assess Job demands, control, support. Responses were given on a four-point Likert scale, from 1 = do not agree to 4 = agree entirely. Mean scores were calculated for job demands, control and support and used as continuous variables. Self-Rated Health (SRH) was asked for in STAGE with the question “How would you rate your general health status?” with response alternatives excellent, good, moderate, fairly poor, and poor. With few responses in the lowest categories, ‘fairly poor’ and ‘poor’ were collapsed into one category. Previous sick leave was based on MiDAS data (episodes of SA > 14 days in a row) between 2003 and STAGE response (approximately a 2-year period before baseline data collection) (yes/no). Don’t know/Don’t want to answer were coded as missing.

### Statistical analyses

Logistic regression analyses were used to assess odds ratios (ORs) with 95% confidence intervals (CIs), interaction with sex and occupational sector was tested, to assess the associations between time pressure, sleep problems due to thoughts about work and SA. Analyses were adjusted for the study sample including twin pairs rather than independent individuals using the clustered robust standard error. When analyzing the whole sample, covariates were entered in three blocks: first sociodemographic factors (age, education, marital status) were entered (Adjusted 1), then job demands, control and support were entered (Adjusted 2), and finally previous history of SA and SRH were added (Adjusted 3). Then analyses were repeated (crude and adjusted 1–3) but stratified by main employer during the past three years. Co-twin control (conditional logistic regression) analyses based on same-sex discordant MZ and DZ twin pairs were conducted to adjust for familial (genetics and shared family environment) confounding (Kujala et al. 2002). A twin pair was treated as discordant if only one twin of a pair had incident SA during follow-up. In co-twin control analyses, twins in a pair are optimally matched on genetics (MZ 100% and DZ on average 50%) and shared environmental factors (100%) when reared together, and for age and sex. An influence of familial factors is indicated if an association found in the whole sample disappears or changes considerably in the analyses of discordant twin pairs. If the association is stronger in DZ than MZ pairs, genetics rather than shared environmental factors are of importance, while familial factors will be assumed to play a minor role if the association is found in the analyses of both the whole sample and of discordant twin pairs. Co-twin analyses were conducted both with MZ and DZ pairs combined and stratified by zygosity. All analyses were conducted using STATA IC 12.1.

## Results

During follow-up 5723 (35%) individuals had an incident SA spell (> 14 days), and a majority of these spells were with full-time benefits. Table 1 presents descriptive statistics of exposures and covariates stratified by SA status. Table 2 shows crude and adjusted ORs for the associations between time pressure, sleep problems due to thoughts about work, and future SA in the whole cohort. A significant association between time pressure and SA were found in the crude analysis only, while the ORs were significant in all adjusted models for sleep problems and SA (ORs 1.22–1.35). There was no interaction with sex. Results by occupational sector showed that the crude estimate of the association between time pressure and SA was significant in the private sector only. Sleep problems due to thoughts about work were associated with future SA among those who had mainly, over the past 3 years, been employed by the state or private sector (ORs 1.20–1.54), see Table 3. However, the only significant interaction was found for county council (*p* = 0.03), but this was no longer statistically significant after adjustment for covariates. The conditional analyses of discordant twin pairs showed estimates in the same direction as for the whole cohort. This indicates no influence of familial factors on the associations studied (Table 4).

**Table 1:** Descriptive statistics of the 16,127 Swedish twin individuals included in the study population by sickness absence (SA) status during follow-up

	No sick-leave *N* = 10,404	Sick-leave *N* = 5723
Exposures	*n* (%)	*n* (%)
Time pressure		
Twice a month or less	6091 (58.5)	3474 (60.7)
Once a week or more	3579 (34.4)	1795 (31.4)
Missing	734 (7.1)	454 (7.9)
Sleep problems		
Twice a month or less	8910 (85.6)	4679 (81.8)
Once a week or more	1440 (13.8)	999 (17.5)
Missing	54 (0.5)	45 (0.8)
*Covariates*		
Main employer past 3 years		
State	988 (9.5)	479 (8.4)
Municipality	1766 (17.0)	1446 (25.3)
County council	633 (6.1)	456 (8.0)
Private sector	5859 (56.3)	2788 (48.7)
Self-employed	743 (7.1)	294 (5.1)
Other	284 (2.7)	172 (3.0)
Missing	131 (1.3)	88 (1.5)
Sex		
Men	5697 (54.8)	2026 (35.4)
Women	4707 (45.2)	3697 (64.6)
Age (range 19–47, mean (SD))	35 (6.9)	36 (6.9)
Education		
Primary	417 (4.0)	409 (7.1)
Secondary/vocational	4352 (41.8)	2584 (45.2)
Higher education	5104 (49.1)	2317 (40.5)
Missing	531 (5.1)	413 (7.2)
Marital status		
Married/cohabiting	7392 (71.0)	4068 (71.1)
Other	2999 (28.8)	1649 (28.8)
Missing	13 (0.1)	6 (0.1)
Job demands (mean, 1–4 (SD))	2.5 (0.6)	2.4 (0.6)
Control (mean, 1–4)	1.9 (0.5)	2.0 (0.6)
Support (mean, 1–4)	1.6 (0.5)	1.7 (0.5)
Previous history of sick leave		
No	9326 (89.6)	4213 (73.6)
Yes	1078 (10.4)	1510 (26.4)
Self-rated health		
Excellent	3677 (35.3)	1599 (27.9)
Good	5104 (49.1)	2827 (49.4)
Moderate	1239 (11.9)	1022 (17.9)
Fairly poor/poor	133 (1.3)	168 (2.9)
Missing	251 (2.4)	107 (1.9)

**Table 2:** Associations between exposures and sickness absence (SA), odd ratios (OR) with 95% confidence intervals (CI)

Sickness absence				
Time pressure	Crude	Adjusted 1	Adjusted 2	Adjusted 3
	OR (95% CI)	OR (95% CI)	OR (95% CI)	OR (95% CI)
Twice a month or less	Ref			
Once a week or more	0.88 (0.82–0.94)	1.01 (0.93–1.09)	0.93 (0.85–1.01)	0.94 (0.86–1.03)
Sleep problems				
Twice a month or less	Ref			
Once a week or more	1.32 (1.21–1.44)	1.35 (1.23–1.49)	1.27 (1.14–1.40)	1.22 (1.10–1.36)

*Adjusted 1*: Sex, age, education, marital status; *Adjusted 2*: Sex, age, education, marital status, job Demands, control, support; *Adjusted 3*: Sex, age, education, marital status, job Demands, control, support, SRH, previous SA

**Table 3:** Associations between time pressure, sleep problems due to thoughts about work and sickness absence (SA) among 16,127 twins, odd ratios (OR) with 95% confidence intervals (CI), by main occupational sector (main employer) the past three years

Sickness absence	Time pressure	Sleep problems
Main employer past 3 years	OR (95% CI)	OR (95% CI)
State	0.95 (0.75–1.20)	1.70 (1.30–2.24)
Municipality	0.92 (0.78–1.08)	1.38 (1.13–1.68)
County council	0.92 (0.71–1.20)	1.02 (0.71–1.47)
Private sector	0.88 (0.79–0.97)	1.31 (1.16–1.49)
Self-employed	1.22 (0.92–1.61)	1.17 (0.84–1.63)

Sickness absence				
Stratified by main employer	Crude	Adjusted 1	Adjusted 2	Adjusted 3
	OR (95% CI)	OR (95% CI)	OR (95% CI)	OR (95% CI)
*State*				
Time pressure				
Twice a month or less	1	1	1	1
Once a week or more	0.95 (0.75–1.20)	1.16 (0.89–1.51)	1.03 (0.77–1.38)	1.05 (0.77–1.42)
Sleep problems				
Twice a month or less	1	1	1	1
Once a week or more	1.70 (1.30–2.24)	1.69 (1.25–2.27)	1.59 (1.15–2.19)	1.54 (1.11–2.13)
*Municipality*				
Time pressure				
Twice a month or less	1	1	1	1
Once a week or more	0.92 (0.78–1.08)	1.02 (0.85–1.21)	0.91 (0.75–1.11)	0.93 (0.77–1.14)
Sleep problems				
Twice a month or less	1	1	1	1
Once a week or more	1.38 (1.13–1.68)	1.43 (1.16–1.76)	1.33 (1.07–1.65)	1.26 (1.00-1.58)
*County council*				
Time pressure				
Twice a month or less	1	1	1	1
Once a week or more	0.92 (0.71–1.20)	1.06 (0.80–1.40)	0.95 (0.70–1.28)	1.04 (0.76–1.43)
Sleep problems				
Twice a month or less	1	1	1	1
Once a week or more	1.02 (0.71–1.47)	1.11 (0.76–1.62)	1.02 (0.67–1.56)	1.07 (0.68–1.67)
*Private*				
Time pressure				
Twice a month or less	1	1	1	1
Once a week or more	0.88 (0.79–0.97)	0.99 (0.89–1.10)	0.95 (0.84–1.07)	0.95 (0.84–1.08)
Sleep problems				
Twice a month or less	1	1	1	1
Once a week or more	1.31 (1.16–1.49)	1.32 (1.16–1.51)	1.25 (1.09–1.44)	1.20 (1.03–1.38)
*Self employed*				
Time pressure				
Twice a month or less	1	1	1	1
Once a week or more	1.22 (0.92–1.61)	1.32 (0.98–1.79)	0.94 (0.62–1.44)	0.90 (0.58–1.41)
Sleep problems				
Twice a month or less	1	1	1	1
Once a week or more	1.17 (0.84–1.63)	1.20 (0.84–1.71)	1.16 (0.72–1.84)	1.15 (0.70–1.90)

*Adjusted 1* sex, age, education, marital status; *Adjusted 2* sex, age, education, marital status, job demands, control, support. *Adjusted 3* sex, age, education, marital status, job demands, control, support, SRH, previous SA

**Table 4:** Discordant twin pair analyses including same-sex twin pairs, odds ratios (ORs) with 95% confidence intervals (CIs)

	Whole sample, age and sex adjusted	All same-sex pairs (MZ + DZ)	DZ same-sex	MZ
	OR (95% CI)	OR (95% CI)	OR (95% CI)	OR (95% CI)
Time pressure (*n* = 919)^a^				
Twice a month or less	Ref			
Once a week or more	0.92 (0.86–0.99)	0.90 (0.73–1.11)	0.75 (0.56–1.01)	1.10 (0.81–1.49)
Sleep problems (*n* = 1057)^a^				
Twice a month or less	Ref			
Once a week or more	1.31 (1.20–1.43)	1.62 (1.24–2.12)	1.64 (1.12–2.38)	1.60 (1.10–2.35)

*MZ* monozygotic, *DZ* dizygotic twin pairs

^a^*n* = 919–1057 discordant pairs

## Discussion

In this study, we investigated whether time pressure and sleep problems due to thoughts about work were associated with future SA among women and men employed in different sectors, also when adjusting for confounders including familial factors. Along with expectations, sleep problems due to thoughts about work once a week or more often were associated with future SA in the fully adjusted model. However, contrary to expectations, genetic or environmental factors shared by family members did not seem to influence the associations. Other study findings clearly demonstrate that both SA (Gjerde et al. 2013; Svedberg et al. 2012) and sleep (Gasperi et al. 2017; Goel 2017; Åkerstedt et al. 2017) are heritable to some extent. But, the survey question used in the present study differs somewhat from questions used in studies reporting genetic influences. Specifically, our focus on work-related sleep problems, rather than sleep length or sleep quality, may explain the lack of familial confounding. However, the association between sleep problems and SA follows previous research using other measures of sleep disturbances (Lallukka et al. 2013, 2014; Madsen et al. 2016; Westerlund et al. 2008). Modern working life may have blurred the distinction between work and leisure time, which, in turn may influence recovery, including sleep. Furthermore, high work demands may contribute to a sustained activation in the evening and night time, which may have negative health impacts (McEwen and Stellar 1993) and subsequently be associated with SA, particularly without adequate recovery and sleep. In the current study, we adjusted for work-related factors including demands, which somewhat reduced the estimates, but these remained significant. A recent study also found high demands to exacerbate the association of sleep problems with incident SA (Madsen et al. 2016). Our findings align with previous research showing that difficulties to stop thinking about work in the evening, modify the effect of work demands, but still remain the strongest predictor of disturbed sleep (Åkerstedt et al. 2002). Moreover, our findings show that worries about work affecting sleep was more important than time pressure for sickness absence. This has prompted researchers to argue that the ability to recover from stress is an important predictor of health-related outcomes: specifically, inadequate recovery may, due to elevated work stress with worries and thoughts of work contributing a sustained activation and poor sleep, be a pathway for developing ill health. Over time, this may, as our findings suggest, lead to SA for both women and men. Thus, for sickness absence, addressing the importance of recovery and to balance adequately work and leisure time seem more important than reducing time pressure for employees and organizations. Similarly, our findings seem to be independent of occupational sector: even though there were some differences in the estimates, with the highest estimate for state employees, only one interaction effect was significant. However, further research including larger samples, to yield adequate statistical power, is needed to confirm these findings and to explore potential differences when it comes to occupations and positions.

While time pressure causing individuals to miss lunch, work late, or bring home work in some studies have shown a protective effect of SA, we found no statistically significant association between time pressure and SA in the present study, apart from in the crude model. Such differences in study findings may be related to the different measures and questions that have been used, which make comparisons between studies somewhat difficult. Moreover, results of protective effects are more pronounced among the highly educated, among men, and among those with higher positions (Krantz and Lundberg 2006). Similar to sleep problems due to thoughts about work, further research taking into account occupation and position is needed.

This study has several strengths including a large and genetically informed population-based sample, high-quality SA data with complete coverage from a national register and a prospective cohort design. Also, extensive survey data including previously used measures of sleep problems due to thoughts about work, time pressure and relevant confounders were available. A unique strength includes the possibility to control for familial confounding using the discordant twin pairs, i.e. to determine whether an association is likely to reflect a causal relationship (Kujala et al. 2002). However, study limitations should be acknowledged. First, questionnaire data always include some missing data. Yet, in the final sample, those with missing data on sleep problems due to thoughts about work constituted only 0.6% and on time pressure the corresponding percentage was 7.4%. Second, without survey follow-ups, all exposures and covariates were only assessed at a single time-point. Consequently, it is unclear whether reports of time pressure, sleep problems or confounding factors change over time, and if such changes influence the risk of SA. Further, only twins, aged 19–47, born in Sweden were included which reduces generalizability to other groups.

In conclusion, the results show that sleep problems due to thoughts about work is a risk factor for future SA. This aligns with previous research showing that sleep length and disturbances, regardless of the underlying reason, is associated with SA. But, experience of time pressure seems to have no effect on future SA. With familial factors not seeming important for the associations studied, a direct relationship between sleep problems and SA is suggested. Importantly, the inability to stop worrying about work during leisure time may link sleep to SA.

## Key messages

What is already known about this subject?

Various risk factors for sickness absence have been investigated and identified. Research regarding time pressure and sleep disturbances due to thoughts about work is limited and no previous studies have taken genetics and shared environment (familial factors) into account when investigating these associations.

What are the new findings?

Sleep problems due to thoughts about work was associated with future sickness absence. This aligns with previous research showing that sleep length and disturbances, regardless of their underlying reasons, are associated with sickness absence. Self-reported time pressure experiences seem to have no effect on future sickness absence. Familial factors did not influence the associations studied.

How might this impact on policy or clinical practice in the foreseeable future?

The inability to stop worrying about work during sleep merits further attention. Working life has changed over the last decades and the 24 h society allows many to work anywhere at any time. With many employees working around the clock, the risk of sleep problems due to thoughts about work and future sickness absence may increase. Addressing the importance of recovery and to balance adequately work and leisure time seem important for employees, organizations and the society.
